# Accuracy of non-physician health workers in respiratory rate measurement to identify paediatric pneumonia in low- and middle-income countries: A systematic review and meta-analysis

**DOI:** 10.7189/jogh.12.04037

**Published:** 2022-04-23

**Authors:** Ahad M Khan, Anna O’Donald, Ting Shi, Salahuddin Ahmed, Eric D McCollum, Carina King, Abdullah H Baqui, Steve Cunningham, Harry Campbell, RESPIRE Collaboration

**Affiliations:** 1 Projahnmo Research Foundation, Dhaka, Bangladesh; 2 The University of Edinburgh, Edinburgh, UK; 3 Johns Hopkins University School of Medicine, Baltimore, Maryland, USA; 4 Karolinska Institutet, Stockholm, Sweden; 5 Johns Hopkins Bloomberg School of Public Health, Baltimore, Maryland, USA

## Abstract

**Background:**

Non-physician health workers play an important role in identifying and treating pneumonia in children in low- and middle-income countries (LMICs). In this systematic review, we summarized the evidence on whether health workers can accurately measure respiratory rate (RR) and identify fast breathing to diagnose pneumonia in children under five years of age.

**Methods:**

We searched MEDLINE, EMBASE, Web of Science, and Scopus from January 1990 to August 2020 without any language restrictions. Reference lists of included studies were also screened for additional records. Studies evaluating the performance of health workers in measuring RR and/or identifying fast breathing compared to a reference standard were included. The methodological quality of the included studies was assessed using the QUADAS-2 tool. A meta-analysis was conducted to report pooled estimates of sensitivity and specificity. Hierarchical summary receiver operating characteristic curve (HSROC) models were fitted, and subgroup and sensitivity analyses were performed to examine the effects of study variables.

**Results:**

We included 16 studies, eight of which reported the agreement in RR count between health workers and a reference standard. The median agreements were 39%, 47%, and 67% within ±2, ±3, and ±5 breaths per minute, respectively. Among the 16 included studies, we identified 15 studies that reported the accuracy of a health worker classifying breathing into either fast or normal categories compared to a reference standard. The median sensitivity, specificity, accuracy, and kappa value were 77%, 86%, 81%, and 0.75, respectively. Seven studies reporting the accuracy of identifying fast breathing were included in the meta-analysis. The pooled estimates of sensitivity and specificity were 78% (95% CI = 72-82) and 86% (95% CI = 78-91), respectively.

**Conclusions:**

Despite the problematic nature of reference standards and their variability across studies, our review suggests that the health worker performance in accurately counting RR is relatively poor. However, their performance shows reasonable specificity and moderate sensitivity in identifying fast breathing. Improving the detection of fast breathing in children with suspected pneumonia among health workers is an important child health programme objective and should be given appropriate priority.

Pneumonia is one of the leading causes of mortality in children aged below five years worldwide [1]. The overall global incidence of pneumonia is 0.22 (IQR = 0.11-0.51) episodes per child-year [2]. Approximately 68 million pneumonia episodes and 650 000 deaths due to pneumonia were estimated to have occurred in 2016 [3]. There is a notable discrepancy between the incidence of pneumonia in high-income countries, in comparison to low- and middle-income countries (LMICs) [2]. Pneumonia presents a substantial burden on health services and is a major cause of hospital admissions in children [4]. In LMICs, the recognition of pneumonia and care-seeking behaviour is generally poor [5]. An important factor limiting the effective diagnosis and treatment of pneumonia in LMICs is a lower doctor-to-population ratio [6]. Moreover, access to doctors and hospitals is usually more difficult [7,8], and the cost of treatment is often prohibitive for caregivers [9]. Therefore, a significant proportion of pneumonia is diagnosed and treated outside hospitals by non-physician health workers [10]. During household visits or community health centre patient encounters, these health workers apply pragmatic case management algorithms to make decisions on diagnosis, treatment, and referral of children suspected to have pneumonia [11,12]. Community-based management of pneumonia by health workers has had a substantial effect on reducing child mortality [13].

According to the World Health Organisation (WHO) guidelines, pneumonia diagnosis in children is primarily based on increased respiratory rate (RR). The number of breaths is manually counted for 60 seconds using an acute respiratory illness (ARI) timer or a watch and is then classified as fast or normal breathing according to the child’s respective age group [14,15]. The measurement of RR is challenging, however, and is frequently miscounted, often due to the child’s movement or shallow, irregular breathing. Counting of RR is often not done routinely by health workers as it is difficult, time-consuming, and depends on the availability of timers. Moreover, a clear definition of a breath is not available within WHO guidelines [16]. This has implications for the quality of clinical practice, as it can lead to under-diagnosis, misdiagnosis, and insufficient or inappropriate treatment [17-19].

The diagnosis of pneumonia in LMICs largely depends on health workers’ ability to count RR and classify fast and normal breathing accurately. Despite existing literature evaluating the ability of health workers to count and classify fast breathing pneumonia, to our knowledge the evidence has not yet been systematically collated. As the existing literature involves studies with small numbers, a systematic review would allow more robust evidence to inform clinical practice and policy implementation. In this review, we summarized the evidence on whether health workers can accurately measure RR and identify fast breathing in children under five years of age.

## METHODS

We conducted this systematic review following the methodology described in the Handbook for Diagnostic Test Accuracy (DTA) Reviews of Cochrane [20]. We used the Preferred Reporting Items for Systematic Reviews and Meta-analyses (PRISMA) 2020 [21] and the Preferred Reporting Items for Systematic Reviews and Meta-analyses of Diagnostic Test Accuracy Studies (PRISMA-DTA) [22] in reporting our findings. The review protocol was registered with the PROSPERO database (registration number CRD42020211127).

### Population, index test, reference standard, and target condition

The target participants were children under five years of age who had their RR assessed in the community or when attending a health facility. The index test was RR counting and/or fast breathing assessment done manually by non-physician health workers. RR counting and/or fast breathing identification by a human expert or an automated device were considered reference standards. The experts were experienced paediatricians, clinicians, or other persons who were trained in clinical algorithms of pneumonia in children.

### Search strategy

We developed a search strategy using a combination of topic-related medical subject headings (MeSH) and keywords. The key concepts were “pneumonia” AND “respiratory rate” AND “accuracy” AND “children under five years of age”. We comprehensively searched MEDLINE (via Ovid), EMBASE (via Ovid), Web of Science, and Scopus databases. The detailed search strategy used for each database is reported in Table S1 in the **Online Supplementary Document**. Included studies were published between January 1st, 1990, to August 9th, 2020. We sought to identify other potentially relevant studies by subjecting all included studies to a forward citation search and examining their reference lists. There were no restrictions on language in the searches. An expert librarian verified the search strategy.

### Study eligibility

Studies were included if they met the following criteria:

1. Measurement of RR and/or identification of fast breathing were done manually by non-physician health workers.
2. A reference standard was used to evaluate the accuracy of RR and/or identifying cases with fast breathing.
3. Age of the participants was less than five years.
4. Conducted in LMICs. The list of LMICs was obtained from the UN Statistics Division (Table S2 in the **Online Supplementary Document**) [23].

Studies were excluded by the following criteria:

1. Non-human animal subjects, or mechanically ventilated subjects.
2. Information on reference standard was lacking.
3. Health workers used a device other than an ARI timer or a watch to measure RR.
4. Health workers counted RR from videotaped subjects.
5. Disaggregation of data on RR or fast breathing was not possible.
6. Disaggregation of data in under-five children was not possible.

### Study selection and data extraction

We downloaded the literature search results from different databases into the EndNote X9 reference management software. After excluding duplicates, two review authors (AMK and AOD) independently examined the titles and/or abstracts of the identified studies and excluded irrelevant studies. They then independently analysed the full texts of potentially relevant articles according to the pre-specified eligibility criteria. Disagreements were resolved through a discussion between the two reviewers.

The review authors extracted data from studies using a structured checklist (Table S3 in the **Online Supplementary Document**) and entered those into the Microsoft Excel spreadsheet. Any disagreements were resolved through discussion.

### Quality assessment

Both reviewers used the Quality Assessment of Diagnostic Accuracy Studies 2 (QUADAS-2) tool [24] to assess the quality of the included studies. Four domains (i.e., patient selection, index test, reference standard, and flow and timing of the participants) were assessed for risk of bias. There are some core signalling questions under each domain. The answer to each signalling question was “yes”, “no” or “unclear”, and the risk of bias was considered as “low”, “high” or “unclear”. The “unclear” category was used only when insufficient data were reported. Individual domain was considered “low risk” if the answers to all signalling questions were “yes”; “high risk” if at least one answer was “no” in any combination; and “unclear” where at least one answer was “unclear”, the other was “yes” and where no answer was “no” in any combination. Both review authors checked the risk of bias independently and any disagreement was settled through discussion. We entered these data into Review Manager (version 5.3) to create the figure used in this paper.

### Data synthesis and analysis

For the studies reporting agreement of RR counts between health workers and the reference standard, we presented the percentage of agreement and calculated median agreement with the range of values. For the studies reporting accuracy of classifying fast and normal breathing compared to a reference standard, we presented sensitivity, specificity, positive predictive value (PPV), negative predictive value (NPV), accuracy, and kappa value of individual study if data were available, and we calculated median values with ranges.

We performed a meta-analysis with those studies reporting classification of fast and normal breathing where true positive (TP), false positive (FP), false negative (FN), and true negative (TN) data could be retrieved. We estimated sensitivity and specificity with 95% confidence intervals (CI) for each study and presented those in paired forest plots to inspect the study variance. We fitted hierarchical summary receiver operating curve (HSROC) models [25] using user-written modules (metandi, midas) [26,27] in the Stata statistical software (version 16.0) to assess accuracy of fast breathing identification. Heterogeneity among studies was evaluated visually, from coupled forest plot, and statistically, using the *I*-square [28]. We used univariate meta-regression to perform subgroup analyses. The parameters for subgroup analysis were as follows: child age, study setting, fast breathing prevalence in the sample, diagnosing health worker, and timing of RR measurement by index test and reference standard.

We performed a sensitivity analysis restricted to studies where fast breathing was defined using WHO RR thresholds. We did not conduct tests for reporting bias due to the ambiguity of the factors of publication bias for diagnostic accuracy studies and the inadequacy of tests for identifying asymmetry of a funnel plot [29].

## RESULTS

### Result of the search

**Figure 1:**
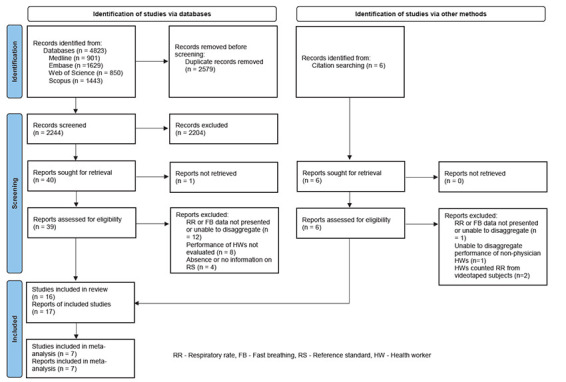
PRISMA flow diagram.

The review process is summarised in **Figure 1** using the PRISMA flowchart [21]. 17 reports with 16 studies met all the criteria for inclusion in this review [17-19,30-43]. Two reports used the same data set but were both included, as they measured different outcomes [35,36]. Only seven studies reporting accuracy of classifying fast and normal breathing presented TP, FP, TN, and FN data, and those studies were included in the meta-analysis [17,18,31,33,35,42,43]. The list of excluded reports with exclusion reasons is available in Table S4 in the **Online Supplementary Document**.

### Characteristics of the included studies

**Table 1** summarises the characteristics of the included studies. Most studies were conducted in Africa [17-19,30,32,33,35-43], two in Asia [30,31] and one in Oceania [34]. 10 studies were based at a health facility [18,30,33,34,37-42], while five were in the community [19,31,32,35,36,43], and one was in a training centre [17]. Studies differed in assessed population, with nine studies assessing children aged 2-59 months [19,32,35,36,38-43], two studies assessed only young infants [31,33], and the remaining studies assessing children varying from 0 to 59 months of age [17,18,30,34,37]. The clinical encounters recorded per study ranged from 34 to 564. The majority of the studies evaluated community-based health workers [17-19,30-32,35-39,43], while three studies evaluated facility-based health workers [40-42] and one study evaluated both [34]. The number of health workers per study ranged from 6 to 154. In most of the studies, the health workers received training before starting the study. The duration of the training ranged from two days to nine months. Only one study used an automated method – the Masimo Root patient monitoring and connectivity platform with ISA CO_2_ Capnography – to measure RR as the reference standard [30]. The remaining studies used a manual count done by an “expert”.

**Table 1:** Characteristics of the included studies

Author, Year	Country	Setting	Population		Index test			Reference standard		Target condition
**Age (months)**	**Observations**	**Performed by**	**Number of HWs**	**Training**	**Performed by**	**Timing**
Baker, 2019 [30]	Cambodia, Ethiopia	Health facility	0-59	322	CHW	Not reported	2 d	Capnography (automated method)	Simultaneously	RR agreement (±2bpm); FB (WHO criteria)
Baqui, 2009 [31]	Bangladesh	Community	0-1	288	CHW	41	6 weeks +3 d refresher	Physician	Long delay	FB (WHO criteria)
Baynes, 2009 [32]	Tanzania	Community	2-59	300	CHW	60	9 mo	IMCI expert re-assessor	Short delay	FB (WHO criteria)
Brady, 1993 [33]	Kenya	Health facility	0-3	200	Nursing students and school graduates	6	1 week	Paediatrician	Short delay	FB (≥60bpm)
Brewster, 1993 [34]	Papua New Guinea	Health facility	1-59	223	Nurse and CHW	104	Not reported	Evaluator	Short delay	FB (≥40bpm)
Cardemil, 2012 [35]	Malawi	Community	2-59	382	CHW	131	6 d	Surveyor	Short delay	FB (WHO criteria)
Gilroy, 2013 [36]	Malawi	Community	2-59	43	CHW	131	6 d	Surveyor	Short delay	RR agreement (±2bpm)
Kallader, 2006 [17]	Western Uganda	Training centre	0-59	564	CHW	96	2 d	Study co-ordinator	Simultaneously	RR agreement (±5bpm); FB (WHO criteria)
Kalyngo, 2012 [37]	Eastern Uganda	Health facility	4-59	57	CHW	57	6 d	Medical officer	Simultaneously	RR agreement (±3, ±5 bpm); FB (WHO criteria)
Kelly, 2001 [38]	Kenya	Health facility	2-59	200, 216 and 414	CHW	100, 108 and 114	3 weeks	Study clinician	Short delay	FB (WHO criteria)
Langston, 2019 [39]	Congo	Health facility	2-59	41 & 39	CHW	154	2-3 mo	Clinician	Short delay	RR agreement (±3bpm)
Miller, 2014 [19]	Ethiopia	Community	2-59	130	HEW	Not reported	Not reported	Clinician	Short delay	RR agreement (±2bpm); FB (WHO criteria)
Mukanga, 2011 [18]	Uganda	Health facility	0-59	182	CHW	14	8 d	Paediatrician	Short delay	RR agreement (±2bpm); FB (WHO criteria)
Mulaudzi, 2015 [40]	South Africa	Health facility	2-59	34	Clinic health care worker	Not reported	Not reported	Researcher	Long delay	FB (WHO criteria)
Simoes, 1992 [42]	Swaziland	Health facility	2-59	331 and 304	Nursing assistant & nurse	3 and 6	Not reported	Paediatrician	Short delay	FB (WHO criteria)
Simoes, 1997 [41]	Ethiopia	Health facility	2-59	254	Nurse	6	9 d	Paediatrician	Short delay	FB (WHO criteria)
Sinyangwe, 2016 [43]	Zambia	Community	2-59	537	CHW	90	6 d	Video recorded and interpreted by experts	Simultaneously	RR agreement (±2, ±3, ±5 bpm); FB (WHO criteria)

RR – Respiratory rate, FB – Fast breathing, CHW – community health worker, bpm – breath per minute, WHO – World Health Organisation

In four studies, health workers and reference standard counted RR simultaneously [17,30,37,43], while there was a short delay (i.e., reference standard measured RR immediately after health worker assessment) in ten studies [18,19,32-36,38,39,41,42] and a long delay (i.e., reference standard measured RR a few hours after health worker assessment) in two studies [31,40]. Studies differed in outcome assessed, with eight studies reporting the percent agreement of RR measurement [17-19,30,36,37,39,43], two reporting Bland-Altman plot to visualise RR agreement [30,43], and 15 reporting correct classification of fast and normal breathing [17-19,30-35,37,38,40-43]. Out of the eight studies reporting agreement in RR, four defined agreement if the difference in RR was within ±2 breaths per minute (bpm) [18,30,36,43], three within ±3 bpm [37,39,43], and four within ±5 bpm [17,19,37,43]. Among the 15 studies reporting accuracy of fast breathing identification, two studies did not use the WHO RR threshold to classify fast breathing [33,34].

### Methodological quality of included studies

**Figure 2:**
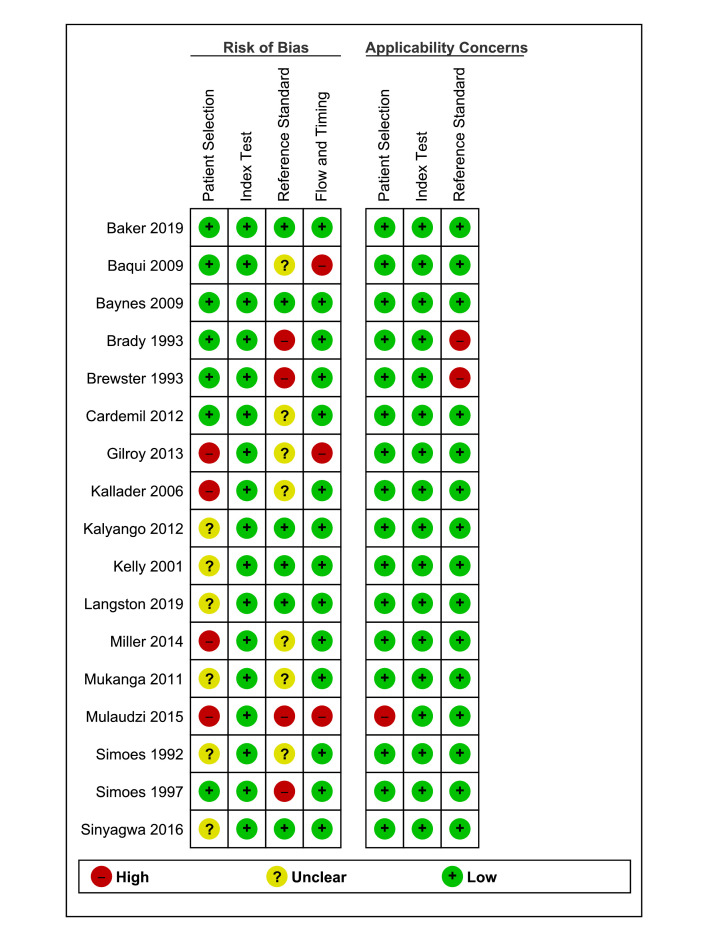
Risk of bias and applicability concerns summary: review authors’ judgements about each domain for each included study.

The assessment of methodological quality is presented in **Figure 2**. In general, the risk of bias was low or unclear. For patient selection, we evaluated four studies as having a high risk of bias because of non-consecutive or non-random sample selection [17,19,36,40], six studies as having unclear risk of bias because of a poorly described sampling method [18,37-39,43] or exclusion criteria [42]. For the index test, we evaluated all studies as having a low risk of bias because the health workers of all studies were blinded to the result of the reference standard, and a pre-specified threshold was used to classify fast breathing. For the reference standard, we evaluated four studies as having a high risk of bias because two studies did not use the WHO RR threshold to classify fast breathing [33,34]. In two studies, reference standard was unblinded [40,41] and seven studies had unclear risk of bias because of poor reporting on blinding [18,19,31,41,42] and qualification of the experts [17,34-36,40]. For patient flow and timing assessment, we deemed three studies to have a high risk of bias. Among these, a long delay between index test and reference standard was present in two studies [31,40], and one study excluded a certain number of patients from the analysis without proper reporting [36]. Most of the studies had low concerns regarding applicability for all domains. The main concerns were related to inclusion criteria for patient selection in one study [40] and inappropriate classification of fast breathing for reference standard in two studies [33,34]. Overall, concerns regarding the applicability of the results were low.

### Agreement in respiratory rate count between health workers and reference standard

**Table 2** presents the summary findings for the eight studies reporting the agreement in RR count between health workers and reference standards. Definitions of agreement in RR count varied across studies. **Table 3** shows that the overall median agreements of the health workers were 39%, 47%, and 67% within ±2 bpm, ±3 bpm, and ±5 bpm of reference standards, respectively. The agreements of RR in terms of age groups, settings, types of health workers, and types of reference standards are also presented.

**Table 2:** Studies reporting agreement in respiratory rate count between health workers and reference standards

Author, year	Included participants	Age (months)	Percent agreement (±2bpm)	Percent agreement (±3bpm)	Percent agreement (±5bpm)
Baker, 2019 [30]	Children with cough or difficult breathing and absence of prolong illness or danger sign presenting at the hospitals	0-2	25/125 = 20%		
		2-59	79/197 = 40%		
		0-59	104/322 = 32%		
Gilroy, 2013 [36]	Children with cough and fast breathing assessed by CHW	2-59	30%		
Kallander, 2006 [17]	Children with fast breathing and children with normal breathing	0-59			409/576 = 71%
Kalyango, 2012 [37]	Children with any acute illness presented at the health facility	4-59		39%	49%
Langston, 2019 [39]	Children with acute respiratory problem presented at hospital	2-59		54% & 49%	
Miller, 2014 [19]	Children with acute illness at rural health posts or household	2-59			91/130 = 70%
Mukanga, 2011 [18]	Children with fever presenting at health centre	2-59	116/182 = 64%		
Sinyangwe, 2016 [43]	Children with suspected pneumonia at the household	2-11	40%	50%	61%
		12-59	48%	56%	69%
		2-59	46%	55%	67%

bpm – breath per minute, CHW – community health worker

**Table 3:** Agreement in respiratory rate count between health workers and reference standards

Characteristics	±2 bpm		±3 bpm		±5 bpm	
**Number of studies**	**Percent agreement (Median and range)**	**Number of studies**	**Percent agreement (Median and range)**	**Number of studies**	**Percent agreement (Median and range)**
**Child age (months)**						
0-2	1	20 (20-20)		-		-
2-59	4	43 (30-64)	2	47 (39-55)	4	60 (49-70)
0-59	1	32 (32-32)			1	71 (71-71)
**Setting**						
Health facility	2	48 (32-64)	1	39 (39-39)	2	51 (49-52)
Community	2	38 (30-46)	1	55 (55-55)	2	69 (67-70)
Training centre		-		-	1	71 (71-71)
**Types of health worker**						
Community-based	4	39 (30-64)	2	47 (39-55)	5	67 (49-71)
Facility-based						-
**Timing of RR measurement**						
Simultaneously	2	39 (32-46)	2	47 (39-55)	3	67 (49-71)
Short delay	2	47 (30-64)		-	2	61 (52-70)
**Types of reference standard**						
Manual count by human	3	46 (30-64)	2	47 (39-55)	5	67 (49-71)
Automated device	1	32 (32-32)				-
**Overall**	4	39 (30-64)	2	47 (39-55)	3	67 (49-71)

bpm – breath per minute

The agreement of RR counts between health workers and a reference standard was presented using the Bland Altman plots in two studies. Baker et al. [30] reported a wide variation in readings, especially in the younger children. The mean difference was -0.6 bpm with limits of agreement (LOAs) from -25.4 to 23.9 bpm [30]. Sinyangwe et al. [43] reported the mean difference of -0.74 bpm with LOAs from -18.8 to 17.3 bpm. Health workers over-counted RR compared to the reference standard in general, but undercounted in children with higher RR.

### Accuracy in fast breathing identification by health workers compared to reference standard

The summary results of the 15 included studies reporting accuracy of classification of fast and normal breathing compared to a reference standard are presented in **Table 4**. The accuracy of fast breathing identification differed in different age groups. The agreement was comparatively lower in children aged 0-2 months compared to older children. The accuracy of fast breathing identification was lower in children with uncomplicated illness, in comparison to children with severe illness.

**Table 4:** Studies reporting health worker classification of fast and normal breathing compared to a reference standard

Author, year	Included participants		Age (month)	Sample prevalence	Sensitivity (95% CI)	Specificity (95% CI)	PPV (95% CI)	NPV (95% CI)	Accuracy (95% CI)	Kappa (SE or 95% CI)
Baker, 2019 [30]	Children with cough or difficult breathing and absence of prolong illness or danger sign presenting at the hospitals		0-<2				0.30 (0.16-0.49)	0.90; (0.82-0.95)		0.26 (0.08)
			2-59				0.42 (0.27-0.56)	0.93 (0.87-0.96)		0.62 (0.07)
			Total				0.53 (0.42-0.64)	0.92 (0.88-0.95)		0.59 (0.05)
Baqui, 2009 [31]	All neonates visited in the households		0-1	0.13	35/40 = 0.88 (0.73-0.96)	243/248 = 0.98 (0.95-0.99)	0.88 (0.74-0.94)	0.98 (0.96-0.99)	0.97 (0.94-0.98)	0.855
Baynes, 2018 [32]	Children with acute illness visited in the households		2-59	0.26					0.81	0.81 (0.78-0.84)
Brady, 1993 [33]	Children with cough, fever or ‘not feeling well’ brought to hospital		0-3	0.20	31/40 = 0.78 (0.62-0.89)	111/160 = 0.69 (0.62-.76)	31/80 = 0.39 (0.32-46)	111/120 = 0.93 (0.87-0.96)	142/200 = 0.71 (0.64-0.77)	
Brewster, 1993 [34]	Children with cough or shortness of breath brought to the facility		1-59	-	19/27 = 0.70		19/22 = 0.86			
Cardemil, 2012 [35]	Children with acute illness presenting to CHW	Uncomplicated illness	2-59	0.18	34/58 = 0.59 (0.46-0.72)	209/256 = 0.82 (0.75-0.88)	34/81 = 0.42 (0.34-0.50)	209/233 = 0.90 (0.86-0.92)	0.77 (0.72-0.82)	0.35 (0.23-0.47)
		Severe illness		0.28	13/19 = 0.68 (0.41-0.95)	42/49 = 0.86 (0.75-0.96)	13/20 = 0.65 (0.46-0.80)	42/48 = 0.88 (0.78-0.92)	0.81 (0.70-0.89)	0.53 (0.31-0.76)
		Overall		0.20	47/77 = 0.61 (0.49-0.72)	251/305 = 0.82 (0.78-0.86)	47/101 = 0.47 (0.39-0.54)	251/281 = 0.89 (0.86-0.92)	0.78 (0.74-0.82)	
Kallander, 2006 [17]	Children with fast breathing and children with normal breathing visited the hospital		0-59	0.48	204/272 = 0.75 (0.69-0.80)	241/292 = 0.83 (0.78-0.87)	204/255 = 0.80 (0.76-0.84)	241/309 = 0.80 (0.75-0.82)	445/564 = 0.79	0.75
Kalyango, 2012 [37]	Children with any acute illness presented at the health facility		4-59						0.75	
Kelly, 2001 [38]	Children with any acute illness presented at hospital	First evaluation	2-59	-	68/110 = 0.62 (0.53-0.70)					
		Second evaluation			74/112 = 0.66 (0.58-0.74)					
		Third evaluation			0.41 (0.31-0.51)					
Miller, 2014 [19]	Children with acute illness at rural health posts or household		2-59						59/94 = 0.63	
Mukanga, 2011 [18]	Children with fever presenting at health centre		2-59	0.35	51/63 = 0.81 (0.69-0.90)	103/119 = 0.87 (0,79-0.92)	51/16 = 0.76 (0.67-0.84)	103/115 = 0.90 (0.84-0.93)	0.85 (0.79-0.90)	
Mulaudzi, 2015 [40]	Children with cough or difficult breathing referred from primary health centre to hospital		2-59				7/14 = 0.50			
Simoes, 1992 [42]	Children with cough or difficult breathing presenting at hospital or clinic	Performed by nursing assistant	2-11	0.33	25/34 = 0.74 (0.56-0.87)	66/70 = 0.94 (0.86-0.98)	25/29 = 0.86 (0.70-0.94)	66/75 = 0.88 (0.81-0.92)	0.88 (0.80-0.93)	
			12-59	0.25	44/57 = 0.77 (0.64-0.87)	140/170 = 0.82 (0.76-0.88)	44/74 = 0.59 (0.51-0.68)	140/153 = 0.92 (0.87-0.95)	0.81 (0.75-0.86)	
			Total	0.27	69/91 = 0.76 (0.66-0.84)	206/240 = 0.86 (0.81-0.90)	69/103 = 0.67 (0.54-0.68)	206/228 = 0.90 (0.87-0.93)	0.83 (0.79-0.87)	
		Performed by nurse	2-11	0.32	26/30 = 0.87 (0.69-0.96)	52/64 = 0.81 (0.70-0.90)	26/38 = 0.68 (0.56-0.79)	52/56 = 0.93 (0.84-0.97)	0.83 (0.74-0.90)	
			12-59	0.26	41/54 = 0.76 (0.62-0.87)	137/156 = 0.88 (0.82-0.93)	41/60 = 0.68 (0.58-0.77)	137/150 = 0.91 (0.87-0.94)	0.85 (0.79 (0.89)	
			Total	0.28	67/84 = 0.80 (0.70-0.88)	189/220 = 0.86 (0.81-0.90)	67/98 = 0.92 (0.88-0.94)	189/206 = 0.84 (0.80-0.88)	0.84 (0.80-0.88)	
Simoes, 1997 [41]	Children with cough or difficult breathing presenting at primary health centre		2-59		0.91	0.89				
Sinyangwe, 2016 [43]	Children with suspected pneumonia at the household		2-11	0.56	67/82 = 0.82 (0.72-89)	53/65 = 0.82 (0.70-0.90)	67/79 = 0.85 (0.77-90)	53/68 = 0.78 (0.69-0.85)	0.82 (0.73-0.88)	0.63
			12-59	0.25	78/98 = 0.80 (0.70-0.87)	236/294 = 0.80 (0.75-0.85)	78/58 = 0.57 (0.51-0.63)	236/256 = 0.92 (0.98-0.95)	0.81 (0.75-0.85)	0.54
			Total	0.34	145/180 = 0.81 (0.74-0.86)	289/357 = 0.81 (0.76-0.85)	145/213 = 0.68 (0.63-0.73)	289/324 = 0.89 (0.86-92)	0.81 (0.76-0.85)	0.59

PPV – positive predictive value, NPV – negative predictive value, CI – confidence Interval, SE – standard error

The median sensitivity, specificity, PPV, NPV, accuracy, and kappa value are presented in **Table 5**. The overall median sensitivity, specificity, and accuracy of classification of fast breathing were 77%, 86%, and 81%, respectively. The median sensitivity was marginally higher in children aged 0-2 months, and median specificity was slightly higher in children aged 2-59 months. The median sensitivity was higher in studies conducted in community settings, whereas the mean specificity was higher in studies conducted in health facilities. Although sensitivities were similar, the specificity was higher in facility-based health workers compared to community-based health workers. The median sensitivity was slightly higher when RR was measured simultaneously by the health worker and reference standard, compared to when it was measured with a short delay. Both median sensitivity and specificity were higher if the prevalence of fast breathing was higher in the sample.

**Table 5:** Health worker classification of fast and normal breathing compared to a reference standard

Characteristics	Median value (range) [number of studies]					
**Sensitivity (%)**	**Specificity (%)**	**PPV (%)**	**NPV (%)**	**Accuracy (%)**	**Kappa**
**Child age (months):**						
0-2	83 (78-88) [n = 2]	80 (69-90) [n = 2]	39 (30-88) [n = 3]	93 (90-98) [n = 3]	84 (71-97) [n = 2]	0.86 (0.86-0.86) [n = 1]
2-59	78 (61-81) [n = 6]	86 (81-91) [n = 6]	68 (42-92) [n = 8]	90 (84-93) [n = 6]	81 (63-84) [n = 8]	0.70 (0.59-0.81) [n = 2]
**Setting:**						
Health facility	77 (62-81) [n = 6]	86 (69-91) [n = 5]	72 (39-92) [n = 8]	90 (84-93) [n = 5]	83 (71-85) [n = 5]	0.59 (0.59-0.59) [n = 1]
Community	81 (61-88) [n = 3]	82 (81-90) [n = 3]	68 (47-88) [n = 3]	89 (89-98) [n = 3]	81 (63-97) [n = 5]	0.81 (0.59-0.86) [n = 3]
Training centre	75 (75-75) [n = 1]	83 (83-83) [n = 1]	80 (80-80) [n = 1]	80 (80-80) [n = 1]	79 (79-79) [n = 1]	0.75 (0.75-0.75) [n = 1]
**Types of health workers:**						
Community-based HWs	78 (61-88) [n = 6]	83 (81-90) [n = 5]	72 (47-88) [n = 6]	90 (80-98) [n = 6]	80 (63-97) [n = 8]	0.75 (0.59-0.86) [n = 5]
Facility-based HWs	78 (76-80) [n = 2]	86 (86-91) [n = 3]	78 (50-92) [n = 4]	87 (84-90) [n = 2]	84 (83-84) [n = 2]	-
**Timing of RR measurement:**						
Simultaneously	78 (75-81) [n = 2]	82 (81-83) [n = 2]	68 (53-80) [n = 3]	89 (80-92) [n = 3]	79 (75-81) [n = 3]	0.59 (0.59-0.75) [n = 3]
Short delay	76 (61-81) [n = 7]	86 (69-87) [n = 5]	72 (39-92) [n = 6]	90 (84-93) [n = 5]	81 (63-85) [n = 7]	0.81 (0.81-0.81) [n = 1]
Long delay	88 (88-88) [n = 1]	90 (90-90) [n = 1]	88 (88-88) [n = 1]	98 (98-98) [n = 1]	97 (97-97) [n = 1]	0.86 (0.86-0.86) [n = 1]
**Types of reference standard:**						
Manual count by human	77 (61-88) [n = 10]	86 (69-91) [n = 9]	76 (39-92) [n = 11]	90 (80-98) [n = 8]	81 (63-97) [n = 11]	0.78 (0.59-0.86) [n = 4]
Automated device	-	-	53 (53-53) [n = 1]	92 (92-92) [n = 1]	-	0.59 (0.59-0.59)
**Prevalence of fast breathing:**						
<median	78 (61-88) [n = 3]	82 (69-90) [n = 3]	47 (39-88) [n = 3]	93 (89-98) [n = 3]	80 (71-97) [n = 4]	0.83 (0.81-0.86) [n = 2]
≥median	80 (75-81) [n = 5]	86 (81-87) [n = 5]	76 (67-92) [n = 5]	89 (80-90) [n = 5]	83 (79-85) [n = 5]	0.67 (0.59-0.75) [n = 2]
**Overall**	77 (61-88) [n = 10]	86 (69-91) [n = 9]	72 (39-92) [n = 12]	90 (80-98) [n = 9]	81 (63-97) [n = 11]	0.75 (0.59-0.86) [n = 5]

PPV – positive predictive value, NPV – negative predictive value

### Results of meta-analysis

**Figure 3:**
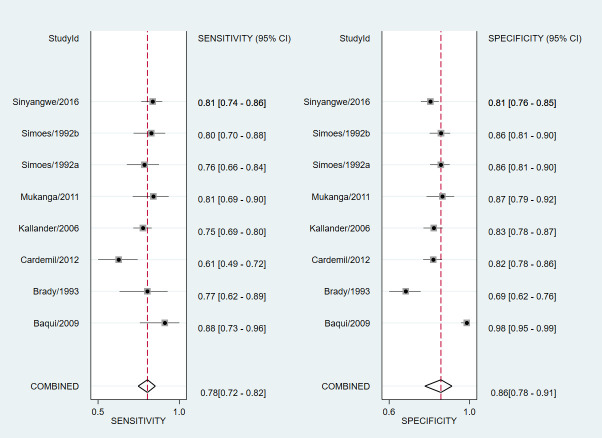
Accuracy of health workers classification of fast and normal breathing compared to a reference standard. Forest plots of individual and summary estimates of sensitivity and specificity.

**Figure 4:**
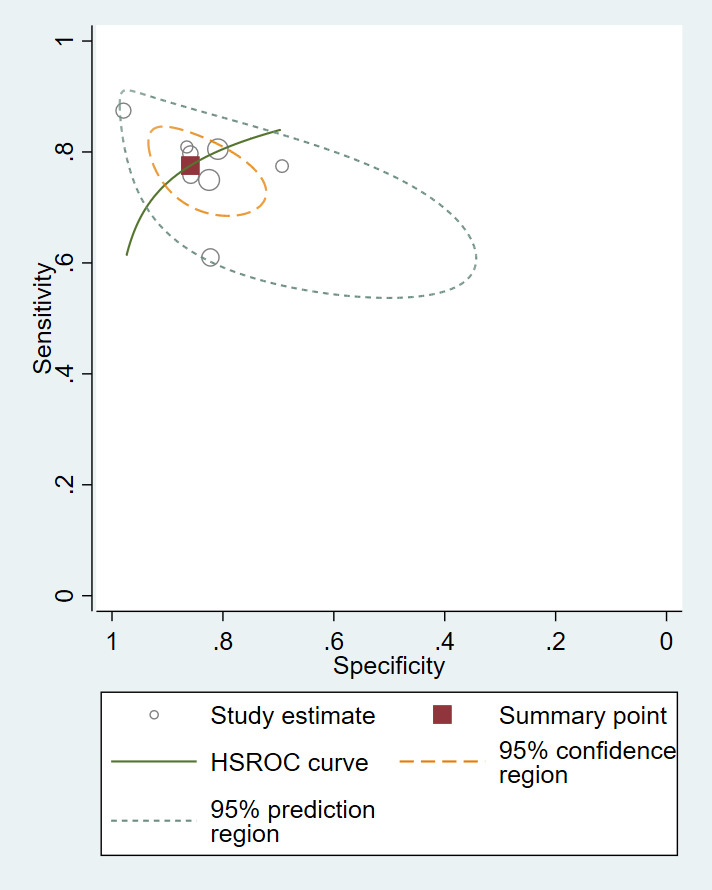
HSROC plot of sensitivity vs specificity of health worker classification of fast and normal breathing for all included studies.

Individual and summary estimates of sensitivity and specificity with 95% CI for all the studies included in the meta-analysis are presented in **Figure 3**. The pooled sensitivity was 78% (95% CI = 72-82), the pooled specificity was 86% (95% CI = 78-91), and there was considerable heterogeneity (*I*^2^ = 72%). **Figure 4** depicts the hierarchical summary receiver curve (HSROC) plot of sensitivity and specificity with summary point, summary estimates, 95% confidence region and 95% prediction region for all studies included in the meta-analysis.

**Table 6** presents subgroup analysis according to child age, study settings, types of health workers, timing of assessment, and prevalence of fast breathing using univariate meta-regression.

**Table 6:** Subgroup analysis of sensitivity and specificity of health worker classification of fast and normal breathing compared to a reference standard

Characteristics	Number of studies	Sensitivity % (95% CI)	Specificity % (95% CI)
**Child age (months):**			
<12	5	0.82 (0.76-0.87)	0.89 (0.81-0.96)
≥12	3	0.78 (0.72-0.84)	0.84 (0.71-0.97)
**Setting:**			
Health facility	5	0.77 (0.71-0.83)	0.83 (0.74-0.92)
Community	3	0.78 (0.70-0.86)	0.90 (0.82-0.97)
**Types of health workers:**			
Community-based	5	0.78 (0.72-0.84)	0.83 (0.74-0.92)
Facility-based	3	0.77 (0.69-0.86)	0.90 (0.82-0.97)
**Timing of RR measurement:**			
Simultaneously	2	0.78 (0.69-0.86)	0.83 (0.74-0.92)
Short or long delay	6	0.77 (0.71-0.83)	0.90 (0.82-0.97)
**Prevalence of fast breathing:**			
<Median	4	0.75 (0.67-0.82)	0.83 (0.74-0.92)
≥Median	4	0.79 (0.73-0.84)	0.90 (0.82-0.97)

CI – confidence Interval, RR – respiratory rate

We conducted a sensitivity analysis excluding the study where the WHO RR threshold was not used to classify fast breathing to explore whether this could affect overall results (Figure S1 in the **Online Supplementary Document**). Based on the studies included in the sensitivity analysis, the pooled sensitivity of fast breathing identification by health workers was 78% (95% CI = 72-83) which was almost similar to the results of the primary meta-analysis (where all studies were included); however, the pooled specificity slightly increased to 87% (95% CI = 81-92).

## DISCUSSION

This systematic review demonstrated that the performance of health workers in the measurement of RR and identification of fast breathing varied across the studies. Overall performance in classifying fast and normal breathing was moderate, with sensitivity ranging from 61% to 88% and a pooled estimate of 78% from the meta-analysis. As the sensitivity is moderate, a significant number of children may have a missed diagnosis of fast breathing, potentially leading to poor outcomes [44]. Some of these children may also have had other clinical signs of respiratory distress, like lower chest wall indrawing, that could have been identified, resulting in a true pneumonia case detection rate higher than these estimates. Further research is needed to investigate possible causes behind the inconsistency in diagnoses between health workers and reference standards, as well as to elicit the difficulties encountered by the health workers, thus improving sensitivity.

The specificity of the studies ranged from 69% to 91%, with a meta-estimate of 86%, demonstrating consistency in exclusion of a diagnosis of fast breathing pneumonia when the disease is not present. This is potentially encouraging, as it may imply that, if these guidelines are followed and RR counting is consistently applied during patient care, then few children would receive antibiotics unnecessarily, which could mitigate inappropriate use of antibiotics [44]. It also means there is minimal unwarranted distress and economic cost for caregivers who would wrongly believe their child has pneumonia [8].

Although there was a moderate agreement in identifying fast breathing, the agreement in RR count between health workers and reference standards was relatively poor. The level of agreement was inconsistent across the studies. The median agreements were 39%, 47%, and 67% within ±2 bpm, ±3 bpm, and ±5 bpm, respectively. It is worth mentioning that obtaining good agreement on RR counts is challenging, even between experts [45]. The difference in RR counts between two observers often does not change the diagnosis. Therefore, classification of RR into fast and normal breathing would be better than the continuous RR count agreements to evaluate the performance of health workers considering its clinical relevance.

The review found that the agreement in RR count between health workers was poor in children aged 0-2 months compared to the older children. The health workers may find it easier to count RR when it is slower in older children compared to when it is fast in younger children [46]. Interestingly, the review found that, although the specificity of fast breathing identification was higher in children aged 2-59 months, the sensitivity was higher in children aged 0-2 months. However, this finding for identifying fast breathing in newborns was based on two studies only. Sensitivity was also found to be slightly higher in infants compared to older children. More studies evaluating the accuracy of RR measurement and fast breathing identification in newborns and infants would be required to confirm this.

Community-based health workers performed better at counting RR and identifying fast breathing compared to facility-based workers. This might be due to community-based workers are usually recruited and trained for a specific program. They usually assess similar signs and symptoms repeatedly, give more time to do an assessment, develop better skills in assessing those specific signs and symptoms, and thus become more experienced, despite being lower cadres [47]. On the other hand, facility-based workers must deal with different types of patients with a wide range of signs and symptoms. The sensitivity was higher in the studies conducted in the community settings compared to those in facility settings. The crowded and busy environment of the health facilities in LMICs might influence the performance of the health workers [48].

The interval between health worker assessment and reference standard assessment is also important in evaluating the performance of health workers. The review demonstrates marginally higher sensitivity when both assessments were done simultaneously compared to a short or long delay. The RR can change over a period of time and this variability may affect sensitivity and specificity in identifying fast breathing [45]. Therefore, simultaneous measurement of RR by a health worker and a reference standard should be ideal. A short delay is not a valid reference standard for comparing RR but may be fair for comparing a binary pneumonia diagnosis. A prolonged period between the two measurements should be avoided.

The absence of an appropriate reference standard to evaluate the performance of health workers is a challenge. Most of the included studies used manual RR count by an expert as the reference standard. An expert is assumed to be more correct. However, the expert can over-count or under-count breaths. Therefore, using expert counting as a reference standard itself poses challenges due to uncertain accuracy. The possible biases using human expert count as the reference standard includes the difficulty in measuring the RR over the same simultaneous period and inconsistencies in human expert RR counting. One study used capnography as reference, which is an automated method using carbon dioxide (CO_2_) in exhaled air to extract RR [49]. However, the validity of using capnography in measuring RR in field-setting is yet to be established. The videography of child assessment and interpretation of the videos by an expert panel could be recommended as a reference standard for future studies [50,51].

There were several limitations to this review. First, most of the studies included in this review were conducted in Africa, while only two were conducted in Asia, and one in Oceania. Therefore, the review findings might not be generalizable across LMICs. Second, RR was often measured by health workers as a part of a larger study. The study may not have provided sufficient information about the methods of measurement and comprehensive results. Third, in most studies, a varying level of training was provided to the health worker before their assessment. This could impact the results of this review [52]. This also raises the question of whether the results of these studies assess health workers’ performance in their day-to-day environments instead of their competency after training. Performance of health workers during the study might not accurately reflect their day-to-day performance; it may also decay over time from training. Fourthly, most of the studies used an expert person as the reference standard who observed the assessment performed by the health workers. The performance of health workers might increase due to the observation compared to when conducting their usual day-to-day activities. This means that the findings would reflect a best-case scenario of accuracy and in the real-world context, we might expect it to be even worse [53]. Fifth, different studies used different definitions of RR agreement, ranging from two to five bpm between health workers and the reference standards. Therefore, it was not possible to combine the findings of all studies that reported agreement of RR measurement. Sixth, we have discussed some factors responsible for the variability of performance of health workers across the studies. There could be quite a few more contributing factors. Finally, we could not include some studies in this review that assessed health workers’ performance in this review, including diagnosis and management of pneumonia, which would involve measuring RR and classifying fast breathing. It was unclear whether these outcomes were measured or measured and not reported. Moreover, we could not include some studies in the meta-analysis because TP, FP, FN, and TN data were missing in the reports, or these were not possible to retrieve.

Despite these limitations, this review provides evidence on the need of strengthening the performance of health workers to measure RR and identify fast breathing pneumonia. Counting RR is the cornerstone to the diagnosis of pneumonia in children, but it is rarely practised in the field during real-world care [54]. The performance of health workers could be enhanced by improved training, supportive supervision, ongoing performance monitoring, and feedback [55]. Counting RR manually is challenging often results in inaccurate diagnosis. Therefore, the development of improved pneumonia diagnostic aids, such as a validated automated RR counters appropriate for use by health workers, might improve the diagnosis of pneumonia in LMICs [56]. Appropriate methods including a non-biased reference standard should be used to evaluate the accuracy of health workers’ RR counts. Further implementation research could help define what the best approach for improving their performance.

## CONCLUSIONS

This review showed that the accuracy of RR measurement by non-physician health workers varied across the studies. While they could measure RR and identify fast breathing pneumonia with a moderate sensitivity and reasonable specificity, there is still a need for the improvement of RR measurement and identification of fast-breathing pneumonia by these health workers. This could be done through improved training, ongoing supervision, audit of performance, and improved diagnostic aids to measure RR and classify fast breathing accurately. The contribution of well-trained and well-equipped health workers is valuable in LMICs, where it is not always feasible for a child to see a doctor. This should decrease the burden on already scarce doctors and health centres in LMICs and may help reduce morbidity and mortality associated with pneumonia.

## Additional material


Online Supplementary Document
