# Supplementary material for: Accuracy of non-physician health workers in respiratory rate measurement to identify paediatric pneumonia in low- and middle-income countries: A systematic review and meta-analysis
Source: J Glob Health. 2022 Apr 23;12:04037. doi: 10.7189/jogh.12.04037 (PMC9037577; doi:10.7189/jogh.12.04037)

Table S1. Search strategies

**A. Name of Database: Ovid MEDLINE(R) 1946 to July Week 5 2020**		
1	exp Pneumonia/	106387
2	Respiratory Tract Infections/	38489
3	pneumon*.tw.	166101
4	(respiratory tract infection* or acute respiratory infection* or acute respiratory tract infection* or arti or lower respiratory infection* or lrti).tw.	23313
5	Infant, Newborn, Diseases/	37453
6	(child* illness* or newborn illness* or neonatal illness* or newborn infection* or neonatal infection*).tw.	4965
7	1 or 2 or 3 or 4 or 5 or 6	293794
8	Respiratory Rate/	2841
9	Respiration/	78270
10	(respiratory rate or breath* rate* or breath* count*).tw.	12849
11	(fast breath* or tachypn?ea).tw.	3672
12	((pneumon* adj3 diag*) or (pneumon* adj3 class*) or (illness* adj3 class*) or (sick* adj3 class*) or ("very severe disease*" adj3 class*)).tw	7579
13	8 or 9 or 10 or 11 or 12	99317
14	exp Child/	1908346
15	exp Infant/	1138346
16	exp Infant, Newborn/	607625
17	exp Pediatrics/	58180
18	(child* or infant* or p?ediatric* or neonat* or newborn*).tw.	1819066
19	14 or 15 or 16 or 17 or 18	2920174
20	"Sensitivity and Specificity"/	347109
21	(accura* or correct* or valid* or reliab*).tw.	1810052
22	(sensitiv* or specific* or "positive predictive value*" or ppv or "negative predictive value*" or npv).tw.	3555492
23	(performance or agreement).tw.	919530
24	20 or 21 or 22 or 23	5524481
25	7 and 13 and 19 and 24	1006
26	limit 25 to humans	996
**27**	**limit 26 to yr="1990 -Current"**	**901**
**B. Name of Database: Embase 1980 to 2020 Week 32**		
1	exp pneumonia/	294581
2	exp respiratory tract infection/	384324
3	pneumon*.tw.	253484
4	(respiratory tract infection* or acute respiratory infection* or acute respiratory tract infection* or arti or lower respiratory infection* or lrti).tw.	38011
5	newborn disease/	18198
6	infant disease/	6416
7	childhood disease/	58251
8	(child* illness* or newborn illness* or neonatal illness* or newborn infection* or neonatal infection*).tw.	6810
9	1 or 2 or 3 or 4 or 5 or 6 or 7 or 8	739298
10	breathing rate/	38773
11	(respiratory rate or breath* rate* or breath* count*).tw.	21825
12	(fast breath* or tachypn?ea).tw.	7264
13	((pneumon* adj3 diag*) or (pneumon* adj3 class*) or (illness* adj3 class*) or (sick* adj3 class*) or ("very severe disease*" adj3 class*)).tw	12325
14	10 or 11 or 12 or 13	63596
15	child/	1620027
16	exp infant/	924397
17	newborn/	488454
18	pediatrics/	72573

19	(child* or infant* or p?ediatric* or neonat* or newborn*).tw.	2393684
20	15 or 16 or 17 or 18 or 19	3107856
21	"sensitivity and specificity"/	363805
22	diagnostic accuracy/	253352
23	predictive value/	173413
24	accuracy/	166097
25	validation study/	84471
26	(accura* or correct* or valid* or reliab*).tw.	2884256
27	(sensitiv* or specific* or "positive predictive value*" or ppv or "negative predictive value*" or npv).tw.	4962706
28	(performance or agreement).tw.	1420506
29	21 or 22 or 23 or 24 or 25 or 26 or 27 or 28	8087311
30	9 and 14 and 20 and 29	1754
31	limit 30 to human	1667
**32**	**limit 31 to yr="1990 -Current"**	**1629**
**C. Name of database: Web of Science Core Collection (1990-current)**		
1	TOPIC: (pneumon*)	198702
2	TOPIC: ("respiratory tract infection*" OR "acute respiratory infection*" OR "acute respiratory tract infection*" OR arti OR "lower respiratory infection*" OR lrti)	25020
3	TOPIC: ("child* illness*" OR "newborn illness*" OR "neonatal illness*" OR "newborn infection*" OR "neonatal infection*")	4165
4	#3 OR #2 OR #1	207847
5	TOPIC: ("respiratory rate*" OR "breath* rate*" OR "breath* count*")	14943
6	TOPIC: ("fast breath*" OR tachypn?ea)	838
7	TOPIC: ((pneumon* NEAR/3 diag*) OR (pneumon* NEAR/3 class*) OR (illness* NEAR/3 class*) OR (sick* NEAR/3 class*) OR ("very severe disease*" NEAR/3 class*))	8639
8	#7 OR #6 OR #5	24142
9	TOPIC: (child* OR infant* OR p$ediatric* OR neonat* OR newborn*)	241336
10	TOPIC: (accura* OR correct* OR valid* OR reliab*)	5252907
11	TOPIC: (sensitiv* OR specific* OR "positive predictive value*" OR ppv OR "negative predictive value*" OR npv)	6087810
12	TOPIC: (performance OR agreement)	5047872
13	#12 OR #11 OR #10	13674801
**14**	**#4 AND #8 AND #9 AND #13**	**850**
**D. Name of database: Scopus**		
1	TITLE-ABS-KEY (pneumon*)	419494
2	TITLE-ABS-KEY ("respiratory tract infection*" OR "acute respiratory infection*" OR "acute respiratory tract infection*" OR arti OR "lower respiratory infection*" OR lrti)	111608
3	TITLE-ABS-KEY ("child* illness*" OR "newborn illness*" OR "neonatal illness*" OR "newborn infection*" OR "neonatal infection*")	12,662
4	#1 OR #2 OR #3	513166
5	TITLE-ABS-KEY ("respiratory rate*" OR "breath* rate*" OR "breath* count*")	43,689
6	TITLE-ABS-KEY ("fast breath*" OR tachypn?ea)	1,427
7	TITLE-ABS-KEY ((pneumon* W/3 diag*) OR (pneumon* W/3 class*) OR (illness* W/3 class*) OR (sick* W/3 class*) OR ("very severe disease*" W/3 class*))	14,400
8	#5 OR #6 OR #7	58,975
9	TITLE-ABS-KEY (child* OR infant* OR p?ediatric* OR neonat* OR newborn*)	4,109586
10	TITLE-ABS-KEY (accura* OR correct* OR valid* OR reliab*)	7,659931
11	TITLE-ABS-KEY (sensitiv* OR specific* OR "positive predictive value*" OR ppv OR "negative predictive value*" OR npv)	8,919061
12	TITLE-ABS-KEY (performance OR agreement)	6,846607
13	#10 OR #11 OR #12	19,696016
14	#4 AND #8 AND #9 AND #13	1,539
**15**	**limit #14 to (year="1990 -Current")**	**1,443**

Table S2. List of low- and middle-income country

Serial No	County
1.	Afghanistan
2.	Albania
3.	Albania
4.	American Samoa
5.	Angola
6.	Armenia
7.	Azerbaijan
8.	**Bangladesh**
9.	Belarus
10.	Belize
11.	Benin
12.	Bhutan
13.	Bolivia
14.	Bosnia and Herzegovina
15.	Botswana
16.	Brazil
17.	Bulgaria
18.	Burkina Faso
19.	Burundi
20.	Cabo Verde
21.	**Cambodia**
22.	Cameroon
23.	Central African Republic
24.	Chad
25.	China
26.	Colombia
27.	Comoros
28.	Democratic Republic of Congo OR DR Congo OR DRC OR the Congo
29.	**Congo**, Rep.
30.	Costa Rica
31.	Côte d'Ivoire OR Ivory Coast
32.	Cuba
33.	Djibouti
34.	Dominica
35.	Dominican Republic
36.	Ecuador

37.	Egypt, Arab Rep.
38.	El Salvador
39.	Equatorial Guinea
40.	Eritrea
41.	**Ethiopia**
42.	Fiji
43.	Gabon
44.	The Gambia
45.	Georgia
46.	Ghana
47.	Grenada
48.	Guatemala
49.	Guinea
50.	Guinea-Bissau or Guinea Bissau
51.	Guyana
52.	Haiti
53.	Honduras
54.	India
55.	Indonesia
56.	Iran, Islamic Rep.
57.	Iraq
58.	Jamaica
59.	Jordan
60.	Kazakhstan
61.	**Kenya**
62.	Kiribati
63.	Korea, Dem. People's Rep.
64.	Kosovo
65.	Kyrgyz Republic
66.	Lao PDR
67.	Lebanon
68.	Lesotho
69.	Liberia
70.	Libya
71.	Macedonia, FYR
72.	Madagascar
73.	**Malawi**
74.	Malaysia
75.	Maldives

76.	Mali
77.	Marshall Islands
78.	Mauritania
79.	Mauritius
80.	Mexico
81.	Micronesia, Fed. Sts.
82.	Moldova
83.	Mongolia
84.	Montenegro
85.	Morocco
86.	Mozambique
87.	Myanmar
88.	Namibia
89.	Nauru
90.	Nepal
91.	Nicaragua
92.	Niger
93.	Nigeria
94.	Pakistan
95.	**Papua New Guinea**
96.	Paraguay
97.	Peru
98.	Philippines
99.	Romania
100.	Russian Federation
101.	Rwanda
102.	Samoa
103.	São Tomé and Principe OR Timor OR Timor-Liste OR Timor Liste
104.	Senegal
105.	Serbia
106.	Sierra Leone
107.	Solomon Islands
108.	Somalia
109.	**South Africa**
110.	South Sudan
111.	Sri Lanka
112.	Saint Lucia
113.	Vincent and the Grenadines
114.	Sudan

115.	Suriname
116.	**Swaziland**
117.	Syrian Arab Republic
118.	Tajikistan
119.	**Tanzania**
120.	Thailand
121.	Timor-Leste
122.	Togo
123.	Tonga
124.	Tunisia
125.	Turkey
126.	Turkmenistan
127.	Tuvalu
128.	**Uganda**
129.	Ukraine
130.	Uzbekistan
131.	Vanuatu
132.	Venezuela, RB
133.	Vietnam
134.	West Bank and Gaza
135.	Yemen, Rep.
136.	**Zambia**
137.	Zimbabwe

Table S3. Extraction of data from eligible studies

**A. Identification of the study**		
1	Study ID	
2	Title	
3	First author	
4	Year of publication	
5	Name of the journal	
6	Study Region – Country	
**B. Study methods**		
7	Study Settings – Facility/community	
8	Study period	
9	Sampling method	
**C. Population characteristics**		
10	Age group	
11	Number of children	
12	Number of observations	
13	Inclusion criteria	
14	Exclusion criteria	
**D. Index test**		
15	Index test used	
16	Number of health workers	
17	Education	
18	Training	
19	Blinding	
**E. Reference standard**		
20	Reference standard used	
21	Qualification	
22	Blinding	
**F. Respiratory rate assessment**		
23	Device used	
24	Sequence of assessment	
25	Time interval between index test and reference standard	
26	Definition of agreement of respiratory rate count	
27	Definition of fast breathing	
**G. Study findings**		
Respiratory rate measurement		
28	Percent agreement of RR counts	
Fast breathing identification		
29	Prevalence of fast breathing in the sample	
30	Kappa value	

31	True positive	
32	False positive	
33	False negative	
34	True negative	
35	Sensitivity (95% CI)	
36	Specificity (95% CI)	
37	Positive predictive value (95% CI)	
38	Negative predictive value (95% CI)	
39	Accuracy (95% CI)	
40	Notes	

Table S4. Excluded studies with reasons for exclusion

Sl	Author, Year	Title	Reason for exclusion
1	Aftab et al., 2018	Improving community health worker performance through supportive supervision: a randomised controlled implementation trial in Pakistan	No information of reference standard
2	Bandyopadhyay et al., 2003	Are primary health workers skilled enough to assess the severity of illness among young infants?	Could not disaggregate respiratory rate or fast breathing data
3	Biswas et al., 2011	Skill of frontline workers implementing integrated management of neonatal and childhood illness: experience from a district of west Bengal, India	Could not disaggregate respiratory rate or fast breathing data
4	Bjornstad et al., 2014	Determining the quality of IMCI pneumonia care in Malawian children	Could not disaggregate respiratory rate or fast breathing data
5	Bruce et al., 2007	Pneumonia case-finding in the RESPIRE Guatemala indoor air pollution trial: standardizing methods for resource-poor settings	Could not disaggregate respiratory rate or fast breathing data
6	Darnstadt et al., 2009	Validation of community health workers' assessment of neonatal illness in rural Bangladesh	Could not disaggregate respiratory rate or fast breathing data
7	Elimian et al., 2020	Comparing the accuracy of lay diagnosis of childhood malaria and pneumonia with that of the revised IMCI guidelines in Nigeria	Caregiver performance was evaluated
8	Gadomski et al., 1993	Caretaker recognition of respiratory signs in children: Correlation with physical examination findings, X-ray diagnosis and pulse oximetry	Caregiver performance was evaluated
9	Getachew et al., 2019	Health Extension Workers' diagnostic accuracy for common childhood illness in four regions of Ethiopia: a cross-sectional study	Could not disaggregate respiratory rate or fast breathing data
10	Gove et al., 1997	Integrated management of childhood illness: field test of the WHO/UNICEF training course in Arusha, United Republic of Tanzania	Could not disaggregate respiratory rate or fast breathing data
11	Hadi et al, 2003	Management of acute respiratory infections by community health volunteers: experience of Bangladesh Rural Advancement Committee (BRAC)	Could not disaggregate respiratory rate or fast breathing data
12	Hadi et al., 2001	Diagnosis of pneumonia by community health volunteers: experience of BRAC, Bangladesh	Could not disaggregate respiratory rate or fast breathing data
13	Hazir et al., 2013	Measuring coverage in MNCH: A prospective validation study in Pakistan and Bangladesh on measuring correct treatment of childhood pneumonia	Health worker performance was not evaluated
14	Huicho et al., 2008	How much does quality of childcare vary between health workers with differing durations of training? An observational multicounty study	Health worker performance was not evaluated
15	Kahigwa et al., 2002	Inter-observer variation in the assessment of clinical signs in sick Tanzanian children	Absence of reference standard
16	Kambarami et al., 1996	Ability of caregivers to recognise signs of pneumonia in coughing children aged below five years	Caregiver performance was evaluated
17	Lanaspa et al., 2014	High Reliability in Respiratory Rate Assessment in Children with Respiratory Symptomatology in a Rural Area in Mozambique	Absence of reference standard
18	Mayhew et al., 2015	Long and short integrated management of childhood illness (IMCI) training courses in Afghanistan: a cross sectional cohort comparison of post-course knowledge and performance	Could not disaggregate respiratory rate or fast breathing data

Sl	Author, Year	Title	Reason for exclusion
19	Mengistu et al., 2014	Effect of performance review and clinical mentoring meeting (PRCMM) on recording of community case management by health extension workers in Ethiopia	Could not disaggregate respiratory rate or fast breathing data
20	Muro et al., 2017	Variability of respiratory rate measurements in children suspected with non-severe pneumonia in north-east Tanzania	Agreement between two paediatricians was evaluated
21	Sarswati et al., 2020	'Know-Can' gap: gap between knowledge and skills related to childhood diarrhoea and pneumonia among frontline workers in rural Uttar Pradesh, India	Use of videotape to assess performance of health workers
22	Sgewade et al., 2013	Integrated Management of Neonatal and childhood illness (IMNCI): Skill assessment of health and integrated child development scheme (ICDS) workers to classify sick under-five children	Could not disaggregate respiratory rate or fast breathing data
23	Simoes et al., 1991	Respiratory rate: measurement of variability over time and accuracy at different counting periods	Performance of health workers was not evaluated
24	Singhi et al., 2003	Counting respiratory rate infants under 2 months: comparison between observation and auscultation	Absence of reference standard
25	Uwemedimo et al., 2018	Distribution and determinants of pneumonia diagnosis using Integrated Management of Childhood illness guidelines: a nationally representative study in Malawi	Unable to disaggregate performance of non-physician health workers
26	Weber et al., 1996	Evaluation of an algorithm for the integrated management of childhood illness in an area with seasonal malaria in the Gambia	Could not disaggregate respiratory rate or fast breathing data
27	Yalcin et al., 2018	Agreement between integrated management of childhood illness and final diagnosis in acute respiratory tract infections	Performance of health workers was not evaluated
28	Zeitz et al., 1993	Community Health worker competency in managing acute respiratory infections of childhood in Bolivia	Use of videotape to assess performance of health workers

Figure S1. Sensitivity analysis of health worker classification of fast and normal breathing compared to reference standard with the studies which used the WHO respiratory rate threshold of fast breathing identification.

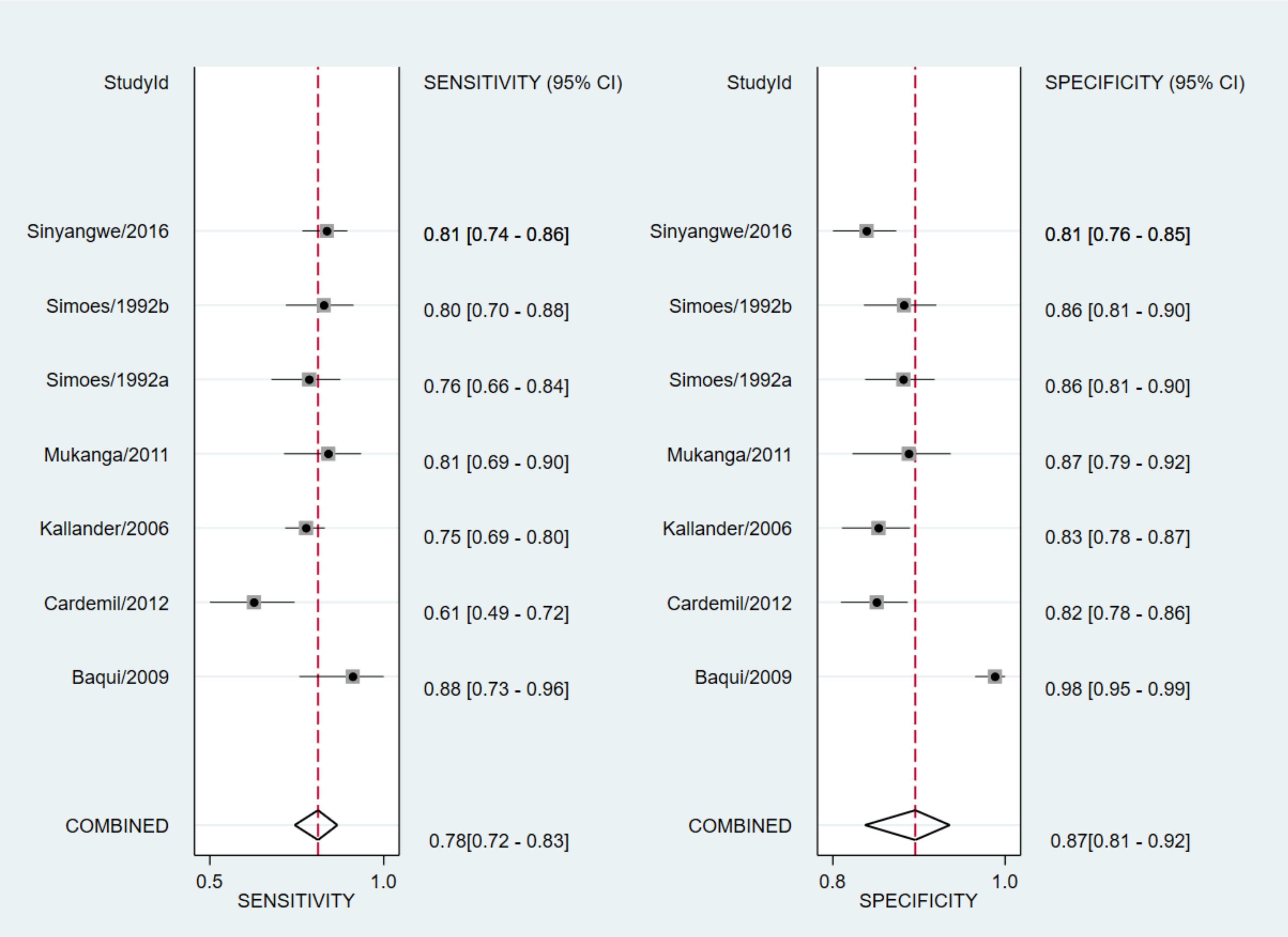

Supplement: Online Supplementary Document [file jogh-12-04037-s001.pdf]
